# Predicting Treatment Response in Schizophrenia With Magnetic Resonance Imaging and Polygenic Risk Score

**DOI:** 10.3389/fgene.2022.848205

**Published:** 2022-02-02

**Authors:** Meng Wang, Ke Hu, Lingzhong Fan, Hao Yan, Peng Li, Tianzi Jiang, Bing Liu

**Affiliations:** ^1^ Brainnetome Center and National Laboratory of Pattern Recognition, Institute of Automation, Chinese Academy of Sciences, Beijing, China; ^2^ School of Artificial Intelligence, University of Chinese Academy of Sciences, Beijing, China; ^3^ Center for Excellence in Brain Science and Intelligence Technology, Chinese Academy of Sciences, Shanghai, China; ^4^ Peking University Sixth Hospital/Institute of Mental Health, Beijing, China; ^5^ Key Laboratory of Mental Health, Ministry of Health (Peking University), Beijing, China; ^6^ Key Laboratory for NeuroInformation of Ministry of Education, School of Life Science and Technology, University of Electronic Science and Technology of China, Chengdu, China; ^7^ Innovation Academy for Artificial Intelligence, Chinese Academy of Sciences, Beijing, China; ^8^ State Key Laboratory of Cognitive Neuroscience and Learning, Beijing Normal University, Beijing, China; ^9^ Chinese Institute for Brain Research, Beijing, China

**Keywords:** schizophrenia, treatment prediction, XGBoost, polygenic risk score, magnetic resonance imaging

## Abstract

**Background:** Prior studies have separately demonstrated that magnetic resonance imaging (MRI) and schizophrenia polygenic risk score (PRS) are predictive of antipsychotic medication treatment outcomes in schizophrenia. However, it remains unclear whether MRI combined with PRS can provide superior prognostic performance. Besides, the relative importance of these measures in predictions is not investigated.

**Methods:** We collected 57 patients with schizophrenia, all of which had baseline MRI and genotype data. All these patients received approximately 6 weeks of antipsychotic medication treatment. Psychotic symptom severity was assessed using the Positive and Negative Syndrome Scale (PANSS) at baseline and follow-up. We divided these patients into responders (*N* = 20) or non-responders (*N* = 37) based on whether their percentages of PANSS total reduction were above or below 50%. Nine categories of MRI measures and PRSs with 145 different *p*-value thresholding ranges were calculated. We trained machine learning classifiers with these baseline predictors to identify whether a patient was a responder or non-responder.

**Results:** The extreme gradient boosting (XGBoost) technique was applied to build binary classifiers. Using a leave-one-out cross-validation scheme, we achieved an accuracy of 86% with all MRI and PRS features. Other metrics were also estimated, including sensitivity (85%), specificity (86%), F1-score (81%), and area under the receiver operating characteristic curve (0.86). We found excluding a single feature category of gray matter volume (GMV), amplitude of low-frequency fluctuation (ALFF), and surface curvature could lead to a maximum accuracy drop of 10.5%. These three categories contributed more than half of the top 10 important features. Besides, removing PRS features caused a modest accuracy drop (8.8%), which was not the least decrease (1.8%) among all feature categories.

**Conclusions:** Our classifier using both MRI and PRS features was stable and not biased to predicting either responder or non-responder. Combining with MRI measures, PRS could provide certain extra predictive power of antipsychotic medication treatment outcomes in schizophrenia. PRS exhibited medium importance in predictions, lower than GMV, ALFF, and surface curvature, but higher than measures of cortical thickness, cortical volume, and surface sulcal depth. Our findings inform the contributions of PRS in predictions of treatment outcomes in schizophrenia.

## 1 Introduction

Pharmacological therapy has long been the cornerstone of schizophrenia management, which aims to relieve psychotic symptoms, such as delusions, hallucinations, and disorganized thinking, et al. (Kane and Correll, 2010; Patel et al., 2014; Tarcijonas and Sarpal, 2019). Whereas, the treatment outcomes of antipsychotic medications generally vary significantly. According to statistics, approximately 10–30% of schizophrenia patients achieve little symptomatic amelioration after receiving multiple trials of typical antipsychotics. Meanwhile, an additional 30–60% of patients with schizophrenia show partial or inadequate improvement in psychotic symptoms (Patel et al., 2014). Further, the long-term disease courses in schizophrenia are even heterogeneous, which are formulated over time (Tarcijonas and Sarpal, 2019). There are twelve treatment trajectories summarized in an over 20-years follow-up study involving more than 500 patients with schizophrenia (Huber et al., 1980). The great variations of treatment outcomes are also confirmed in more recent studies (Carbon and Correll, 2014; Tarcijonas and Sarpal, 2019). Although varying degrees of remission are acquired in a great number of patients with schizophrenia, substantial evidence suggests that antipsychotic medications can lead to various adverse effects (Muench and Hamer, 2010; Patel et al., 2014; Stroup and Gray, 2018). To date, no clinical reliable quantitative markers can be employed to accurately predict the treatment response to antipsychotic medications of a patient with schizophrenia. Therefore, to avert unnecessary side effects, enable early intervention, and adopt appropriate treatments, it is critical to identify prognostic measures that can inform individual treatment outcomes in advance.

Toward this target, considerable efforts are made to identify predictors of antipsychotic treatment outcomes. Recently, magnetic resonance imaging (MRI) has been broadly applied in psychiatry researches, which provides quantitative *in vivo* measures of the brain (Quinlan et al., 2020; Voineskos et al., 2020; Kraguljac et al., 2021). Particularly, one significant area of these applications is the prediction of antipsychotic treatment responses or outcomes in patients with schizophrenia. Overall, a large number of studies focused on structural MRI measures. A longitudinal study of individuals with first-episode schizophrenia reported that the ventricular volume was significantly increased in patients with poor treatment outcomes, which was not observed in better treatment outcome patients and healthy controls (Lieberman et al., 2001). Another independent longitudinal study confirmed this and found schizophrenia patients with poor treatment outcomes had greater lateral ventricular enlargement over time (Ho et al., 2003). In a cross-sectional comparison study, conducted in schizophrenia patients with poor outcomes, favorable outcomes, and healthy individuals, poor outcome patients showed significantly smaller cerebral gray matter particularly in prefrontal regions, and increased volume in the lateral and third ventricles (Staal et al., 2001). A voxel-based comparison analysis of gray matter volume revealed that non-responder schizophrenia patients demonstrated a more severe atrophy pattern than responder patients, particularly in the superior and middle frontal gyri (Quarantelli et al., 2014). Compared with non-resistant schizophrenia patients, treatment-resistant patients showed a significant decrease of thickness in the left dorsolateral prefrontal cortex (Zugman et al., 2013). Cortical gyrification in bilateral insula, left frontal, and right temporal regions were significantly decreased in non-responder patients with first-episode schizophrenia compared with responders (Palaniyappan et al., 2013). Besides, non-responders had smaller thickness in the occipital lobe and smaller asymmetry in the frontal region compared with responders (Szeszko et al., 2012).

In addition to structural MRI, resting-state functional MRI has also been shown to provide prognostic markers. Functional connectivity was one of the most fully investigated measures. Using a seed-based approach, functional connectivity of the striatum with the dorsolateral prefrontal cortex, anterior cingulate, and limbic regions such as the hippocampus and anterior insula, were observed positively correlated with improvement of antipsychotic treatment in patients with first-episode schizophrenia. This relationship was converse when functional connectivity changed to the striatum with the parietal lobe (Sarpal et al., 2015). The prognostic capability of striatal connectivity was also demonstrated in other studies (Sarpal et al., 2016, 2017). Increased functional connectivity in the default mode network (DMN) with the ventromedial prefrontal cortex was found associated with greater efficacy of treatment using olanzapine in schizophrenia (Sambataro et al., 2010). Besides, functional connectivity of the superior temporal cortex was utilized to successfully predict antipsychotic treatment responses in first-episode drug-naïve schizophrenia patients (Cao et al., 2020). Apart from static functional connectivity, dynamic functional connectivity within DMN regions was proved with the most predictive power of symptom change in schizophrenia compared with other common measures (Kottaram et al., 2020). Several other resting-state functional MRI derived measures were also examined to establish their relationships with treatment outcomes in schizophrenia, such as regional homogeneity (Gao et al., 2018) and amplitude of low-frequency fluctuation (Cui et al., 2019).

Schizophrenia is a highly polygenic disorder with thousands of associated risk loci, with mostly small effects (Smeland et al., 2020 ). Polygenic risk score (PRS) is a measure to assess an individual’s genetic liability to schizophrenia, which is calculated by combining total risk alleles with corresponding weights derived from genome-wide association study results (Choi et al., 2020). In a recent study, PRS was verified as a predictor of antipsychotic efficacy in first-episode schizophrenia. Patients with higher PRS tended to be treatment non-responders than those with lower PRS (Zhang et al., 2019). However, it remains unclear whether PRS can markedly improve prognostication on the basis of MRI-derived predictors. If indeed better prediction performance is acquired when combining PRS and neuroimaging predictors, the precedence of the predictive capability of these predictors requires to be investigated.

In the present study, we worked on the problem and hypothesized that PRS can provide additional prognostic power combined with MRI predictors. We collected a total of 57 patients with schizophrenia, which were divided into responders and non-responders according to their 6 weeks of antipsychotic treatment outcomes. Various neuroimaging and PRS features were calculated. We constructed machine learning classifiers with these baseline features to identify responders or non-responders. Particularly, we concentrated on 1) performance comparison of a classifier trained using a combination of MRI and PRS features with a classifier trained using single MRI features; 2) relative importance or contributions of these features to predictions.

## 2 Materials and Methods

### 2.1 Participants and Clinical Assessments

Individuals with schizophrenia (*N* = 97, before screening) were recruited from Peking University Sixth Hospital and Beijing Huilongguan Hospital, whose imaging data were all obtained on a 3.0T Siemens TrioTim MRI scanner. Diagnoses were made by qualified clinicians using the Structured Clinical Interview for DSM-IV. All participants had no history of other DSM-IV Axis I disorders, neurological disorders, cognitive deficits, severe physical diseases, serious head trauma, substance abuse or dependence, and electroconvulsive therapy within the last 6 months. Each individual was treated with only a single second-generation antipsychotic drug, although the specific drug is not totally the same across patients (mainly including risperidone and clozapine). The study was approved by the Medical Research Ethics Committees of the local hospitals. All individuals or their guardians provided written informed consent. Participants were excluded if their clinical assessments at baseline or follow-up were incomplete, or they lacked sMRI, rsfMRI, or genotype data. Quality control (QC) for rsfMRI data was completed by examining the framewise displacement (FD) (Power et al., 2012). Individuals who had a mean FD greater than 0.3 mm were precluded. Besides, subjects were also excluded if they failed to genotyping QC. In total, 57 subjects remained after the screening.

The symptom severity of patients with schizophrenia was evaluated using the Positive and Negative Syndrome Scale (PANSS) (Kay et al., 1987) by trained clinical psychiatrists. Baseline assessments were completed within 1 week of image acquisition. Follow-up assessments were performed after approximately 6 weeks of antipsychotic treatment. Table 1 shows demographics and clinical characteristics.

**TABLE 1:** Demographics and clinical information of participants.

	Individuals with schizophrenia (*N* = 57)		
—	**Responder (*N* = 20)**	**Non-responder (*N* = 37)**	***p* value**
Age (years)	25.22 ± 5.4	28.35 ± 7.3	0.10
Sex (male/female)	7/13	20/17	0.27
PANSS total score at baseline	76.90 ± 8.3	79.21 ± 7.8	0.31
PANSS total score at follow-up	44.15 ± 12.4	65.29 ± 8.1	4.30e-10
Percentage reduction of PANSS total score	71.19 ± 27.1%	28.05 ± 13.2%	1.18e-10
Chlorpromazine equivalent dosage (mg/day)	418.42 ± 280.6	531.03 ± 367.9	0.27

PANSS, positive and negative syndrome scale; Data were shown as mean ± standard deviation.

### 2.2 Image Acquisition and Preprocessing

All images were acquired on a 3.0T Siemens TrioTim scanner. Two-dimension echo-planar imaging (EPI) was used for rsfMRI data with parameters: repetition time (TR) = 2000 ms; echo time (TE) = 30 ms; flip angle (FA) = 90^o^; field of view (FOV) = 220 × 220 mm^2^; matrix size = 64 × 64; voxel dimensions = 3.4375 × 3.4375 × 4.6 mm^3^; 240 volumes, and 33 slices. For T1-weighted (T1w) structural images, three-dimension magnetization-prepared rapid gradient-echo (MPRAGE) sequence was performed with parameters: TR = 2,530 ms; TE = 3.5 ms; FA = 7^o^; inversion time (TI) = 1,100 ms; voxel dimensions = 1 × 1 × 1 mm^3^; matrix size = 256 × 256 × 192.

Preprocessing of rsfMRI data was performed using the BRANT toolkit (Xu et al., 2018, https://github.com/kbxu/brant). In brief, several standardized procedures were carried out, including discarding the first ten timepoints, slice timing correction, realignment, coregistration, spatial normalization to Montreal Neurological Institute (MNI) space, resampling, regressing out nuisances of linear trends, global signal as well as head-motion parameters, and performing temporal band-pass filtering at 0.01–0.08 Hz.

### 2.3 Genotype Data Acquisition and Preprocessing

The procedures of genotype data collection and preprocessing were elaborately described in our previous studies (Liu et al., 2017; Hu et al., 2021). Briefly, for all individuals, ethylene diamine tetraacetic acid (EDTA) anti-coagulated venous blood samples were obtained, from which genomic DNA data were extracted using the EZgene Blood gDNA Miniprep Kit. The whole-genome genotyping was carried out on Illumina Human OmniZhongHua-8 BeadChips with the standard Illumina genotyping protocol.

Genotype processing and QC was implemented using PLINK version 1.07 (Purcell et al., 2007), following the subsequent steps: 1) excluded subjects with missing genotype rates more than 0.05; 2) identified subject pairs with highly similar genotypes and removed the one who had a greater missing genotype rate; 3) removed single nucleotide polymorphisms (SNPs) if their missing genotype rates greater than 0.05, with a minor allele frequency less than 0.01, and significantly deviated from Hardy-Weinberg Equilibrium (*p* < 0.001); 4) used EIGENSTART (Patterson et al., 2006; Price et al., 2006) for principal component analysis (PCA) on linkage disequilibrium (LD) pruned set of autosomal SNPs, which were obtained from LD pruning and removing five long-range LD regions using the HapMap phase three reference data set (Thorisson et al., 2005). Outliers of samples with more than six SD were excluded after achieving 10 principal components; 5) imputation was completed using SHAPEIT (Delaneau et al., 2011) and IMPUTE2(Howie et al., 2009) referred to the 1,000 Genomes phase one dataset. The autosomal SNPs with imputation quality scores greater than 0.8 were further analyzed.

### 2.4 Predictors and Clinical Outcome

We calculated diverse predictors (features) based on imaging and genotype data and divided subjects into responder and non-responder groups according to clinical outcomes.

#### 2.4.1 Responder and Non-responder

For each individual, the clinical outcome was measured by percentage reduction of PANSS total score relative to baseline, which was calculated as follows:

$$\Delta =\frac{{\text{PANSS}}_{\text{baseline}}-{\text{PANSS}}_{\text{followup}}}{{\text{PANSS}}_{\text{baseline}}-30}\times 100\%$$

The subtracted value of 30 in the denominator indicates a minimum score of “no symptoms” assessed using PANSS. We defined an individual as a responder in case that the patient achieved a at least 50% reduction of PANSS total score. Subjects not satisfying this criterion were regarded as non-responders. The cut-off threshold was specified at 50%, given that this value roughly reflects a “much improved” condition for acutely ill and non-refractory patients from a clinical perspective (Leucht et al., 2009). Although the statistical power might be reduced when dichotomizing the continuous clinical outcome, it provides a clear and interpretable measure instead (Lewis, 2004; Kottaram et al., 2020).

#### 2.4.2 Gray Matter Volume

Voxel-based morphometry analysis was performed using the VBM8 toolbox (Matsuda et al., 2012, http://dbm.neuro.uni-jena.de/vbm8/), which runs within the SPM8 software (https://www.fil.ion.ucl.ac.uk/spm/software/spm8/). For each subject, the native T1w image was segmented into tissue images of gray matter, white matter, and cerebrospinal fluid, which were then registered to the standard MNI space through non-linear deformation using the high dimensional DARTEL algorithm (Ashburner, 2007). All non-brain tissues were removed in the process. Smoothing was not applied. Each segmented image had a voxel size of 1.5 mm with a resolution of 121 × 145 × 121. Quality control was completed by displaying slices for segmented images and inspecting sample homogeneity. For each gray matter image, we extracted mean gray matter volumes from each of the brain parcellations defined in the Brainnetome atlas (Fan et al., 2016, https://atlas.brainnetome.org/download.html), resulting in a total of 246 regional values.

#### 2.4.3 Cortical Morphologies

Cortical reconstruction was performed on raw T1w images using FreeSurfer version 6.0 (Dale et al., 1999, https://surfer.nmr.mgh.harvard.edu/fswiki/rel6downloads). For each individual, this process estimated various vertex-based cortical surface morphological measures. Quality control was performed by visually examining any errors in the whole reconstruction process. To precisely match cortical locations among subjects, we aligned each reconstructed cortical surface with the fsaverage template, which had 163,842 vertices per hemisphere. We selected five cortical morphologies in the study, including surface area, curvature, sulcal depth, thickness, and volume. As with GMV, we used the Brainnetome parcellations to extract averaged cortical values, resulting in 210 values for each measure. The atlas is already resampled to fsaverage space. Finally, for each individual, we calculated 210 (number of cortical parcellations) × 5 (number of measures) values in total.

#### 2.4.4 Amplitude of Low-Frequency Fluctuation

ALFF is a rsfMRI measure that quantifies the amplitude of spontaneous low-frequency fluctuations of time series signals (Zang et al., 2007). We used the BRANT toolkit to estimate a voxel-based ALFF map for each individual. To be specific, the fast Fourier transform algorithm was first applied to transform time series into the frequency domain and the corresponding power spectrum was achieved. Next, square root values were calculated at each frequency within the spectrum. ALFF was defined as the mean square root across the frequency range of 0.01–0.08 Hz. The rsfMRI data were not performed temporal band-pass filtering before estimating ALFF maps to avoid possible effects. Finally, each ALFF map was normalized by subtracting the global mean then dividing by the global standard deviation to eliminate inter-subject biases. Likewise, we extracted mean values from ALFF maps based on the Brainnetome atlas and obtained 246 regional values for each individual.

#### 2.4.5 Regional Homogeneity

ReHo measures the similarity between the time series in a given voxel and those in its 26 neighboring voxels based on Kendall’s coefficient of concordance (Zang et al., 2004). It is a reflection of synchronization between the time series of a given voxel and its neighbors. We also used the BRANT toolkit to calculate the ReHo map for each subject. Normalization was performed on each ReHo map by dividing the global mean intensity. As with the ALFF map, for each individual, we extracted 246 values from the ReHo map according to the Brainnetome atlas.

#### 2.4.6 Functional Connectivity

For each subject, whole-brain FCs were calculated based on the Brainnetome atlas. We first extracted the mean time series from each of the 246 brain regions defined in the atlas. Then we calculated Pearson’s correlations between the extracted time series of each region pair. Particularly, there were (246 × 245)/2 = 30,135 unique pairs of regions. We obtained 30,135 FCs for each subject, which was substantially greater than the number of total individuals (*N* = 57). Thus we further performed dimensional reduction by applying PCA on FCs from all subjects and achieved 50 principal components, accounting for 95% amount of variance.

#### 2.4.7 Genetic Characteristics

We calculated step-wise polygenic risk scores (PRSs) for each individual with identical procedures in our prior study (Hu et al., 2021). The PRSs were computed using PLINK version 1.07 (Purcell et al., 2007) and genome-wide association study (GWAS) data from a large number of Chinese individuals (Li et al., 2017). Of note, our study cohort was independent of subjects from the GWAS study, despite they matched in ancestries. We established a list of separate *p*-value threshold ranges to aggregate SNPs. Specifically, we set step lengths of 0.001 and 0.01 for [0, 0.05) and [0.05, 1) intervals, respectively. The left square bracket and the right parenthesis denoted inclusion and exclusion cut-off values, separately. Consequently, there were 145 PRSs computed for each individual with distinct SNP inclusion thresholds: [0, 0.001), [0.001, 0.002), …, [0.049, 0.05), [0.05, 0.06), [0.06, 0.07), …, [0.99, 1).

### 2.5 Classification

We sought to build classification models from a combination of features derived from imaging and genotype data to predict whether a patient with schizophrenia was a responder or a non-responder after receiving 6 weeks of antipsychotic treatment.

#### 2.5.1 Model Building, Training, and Testing

To deal with this prediction problem, we employed extreme gradient boosting (XGBoost) (Chen and Guestrin, 2016) to build binary classifiers to predict individual treatment outcomes. XGBoost is a scalable machine learning system for tree boosting and is publicly available as an open-source package (https://github.com/dmlc/xgboost). We chose the XGBoost method mainly for its significant and broadly recognized impact on various machine learning and data mining challenges (Chen and Guestrin, 2016), as well as its successful applications in brain imaging prediction tasks (Torlay et al., 2017; Sharma and Verbeke, 2020).

We calculated several categories of predictors (features): 1) GMV with 246 regional values, 2) cortical morphologies of surface area, curvature, sulcal depth, thickness, and volume, each of which had 210 values, 3) rsfMRI measures of ALFF (246 values), ReHo (246 values), and FC (50 values), as well as 4) 145 genetic features of PRS. In total, 1983 features were computed. All these categories of features were combined to train XGBoost classifiers. Given the modest sample size of the studied cohort, we applied a leave-one-out cross-validation (LOOCV) strategy to validate classifier performance, which is supposed appropriate for small datasets and used in similar tasks (Cao et al., 2020; Kottaram et al., 2020). Specifically, iteratively held out one subject for validation, and used the rest to train the model until all the subjects were validated once. The eventual result was computed by taking the mean of all the subject validations. Several established measures were calculated for evaluations of classification performance, including accuracy, sensitivity, specificity, F1-score, and area under the receiver operating characteristic curve (ROC-AUC).

It is known XGBoost models tend to contain larger hyperparameter sets compared with basic machine learning classifiers, such as logistic regression, support vector machine, et al. Thus hyperparameter tuning is of great importance to leverage the maximum power of this method. Originally, all parameters were assigned to default values. We tuned one parameter each time and kept the others constant to examine changes in classifier performance as the variation of the specified parameter by performing repetitive LOOCV procedures. In this way, we identified which parameters were relatively important that significantly influenced classifier performance, and which parameters had minor impacts on model performance. We also estimated certain value ranges for each of these crucial parameters. Of note, these value ranges were determined separately, which we considered might constitute a possible optimal searching space. Finally, we concentrated on these significant parameters and performed a fine-grained grid search on the estimated value ranges. Besides, due to the imbalanced sample sizes between responders and non-responders, we calculated the sample weights that were inversely proportional to class frequencies and applied them when fitted models.

#### 2.5.2 Feature Importance

A valuable benefit of using the XGBoost method is that it automatically provides estimates of feature importance from a trained predictive model. Generally, we can directly retrieve importance scores for each feature, which measure how useful or valuable each feature is in the construction of the boosting tree model. The importance can be quantified using several metrics provided by XGBoost, such as gain, coverage, weight, total gain, total coverage. We specified the gain metric for our models, which is supposed as the most relevant attribute to interpret the relative importance of each corresponding feature. A feature is considered more important for generating a prediction if its gain value is higher compared to another feature.

In addition to estimating feature importance through the trained classifier itself, we also evaluated the contributions of feature categories. Specifically, we removed one feature category, such as GMV or cortical thickness, and used all the remaining features to reconstruct predictive models with identical procedures as our main analysis in which all feature categories were used. We determined the contribution of each feature category by evaluating performance change (e.g., accuracy) between each newly built classifier and our main model. If removing a feature category led to a maximum decrease in performance, then this feature category was considered to contribute most to predictions.

## 3 Results

### 3.1 Predicting Treatment Response in Schizophrenia

Individuals with schizophrenia were reasonably defined as responders (*N* = 20) or non-responders (*N* = 37) according to their amelioration degrees of overall symptom severity, which was assessed using PANSS total score, after accepting 6 weeks of antipsychotic medications treatment. The responders and non-responders were matched in age and sex. There were also no significant differences between the two groups in baseline PANSS total score and chlorpromazine equivalent dosage (Table 1). We calculated a multitude of predictors (features), spanning categories of 1) structural imaging (GMV; cortical morphologies of surface area, curvature, sulcal depth, thickness, and volume), 2) functional imaging (ALFF; ReHo; FC), and 3) genetic characteristics (step-wise PRS). Combined with both imaging and genetic features, we constructed binary machine learning classifiers using the XGBoost method to predict individual treatment outcomes (i.e., responder or non-responder). We applied a leave-one-out cross-validation (LOOCV) scheme to validate model performance, and reported several estimated classification metrics to provide a comprehensive evaluation. The XGBoost classifiers were trained with carefully hyperparameters fine-tuning processes. Table 2 shows the optimal hyperparameters set for LOOCV.

**TABLE 2:** Optimal hyperparameters set of XGBoost classifier for leave-one-out cross-validation.

Parameters	Description	Value
n_estimators	Number of boosting rounds	50
max_depth	Maximum tree depth for base learners	2
learning_rate	Boosting learning rate	0.12
booster	Specify which booster to use: gbtree, gblinear, or dart	gbtree
gamma	Minimum loss reduction required to make a further partition on a leaf node of the tree	0.01
subsample	Subsample ratio of the training instance	0.90
colsample_bytree	Subsample ratio of columns when constructing each tree	0.30
colsample_bylevel	Subsample ratio of columns for each level	0.50
colsample_bynode	Subsample ratio of columns for each split	0.30
reg_alpha	L1 regularization term on weights	0.10
reg_lambda	L2 regularization term on weights	1.65
scale_pos_weight	Balancing of positive and negative weights	2.50

Other hyperparameters not listed in the table were set to default values. The description referred to the XGBoost documentation at https://xgboost.readthedocs.io/en/latest/index.html.

**FIGURE 1:**
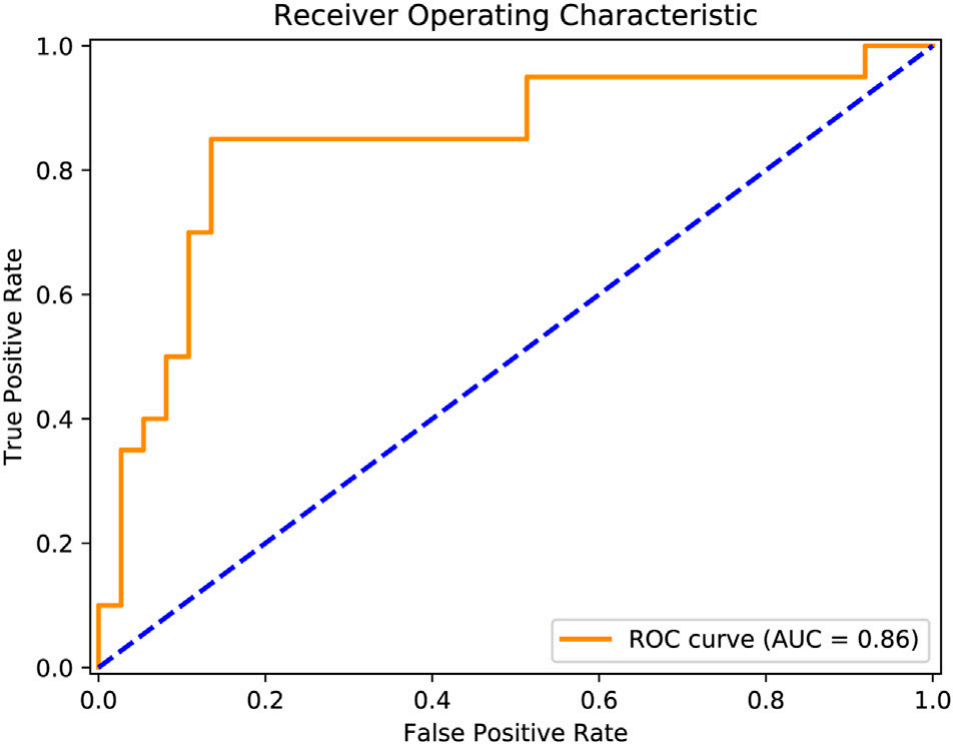
Prediction performance was quantified using the receiver operating characteristic curve. The orange solid line reflected actual classification results, and the blue dashed line indicated the chance level.

We observed the classification accuracy reached a relatively high score of 86% (Table 3). There were eight misclassified individuals altogether, of which 4 subjects were near the cut-off boundary of treatment outcomes (i.e., the 50% threshold). The corresponding percentage reductions of PANSS total score of these four subjects were 45, 43, 40, and 48%. Particularly, several additional metrics that quantify model performance exceeded 80% (Table 3), including sensitivity (85%), specificity (86%), F1-score (81%), ROC-AUC (0.86). Meanwhile, the ROC curve demonstrated our classification results were far higher than the chance level (Figure 1). Taken together, our classifiers had high predictive power and were not biased to a certain class.

**TABLE 3:** Performance of predicting individual treatment outcomes with all imaging and genetic features.

Performance metrics	Accuracy (%)	Sensitivity (%)	Specificity (%)	F1-score (%)	ROC-AUC
Classification results	85.96	85	86.49	80.95	0.86

ROC-AUC, area under the receiver operating characteristic curve. Responder/non-responder = 20/37.

### 3.2 Evaluating Feature Contributions

**FIGURE 2:**
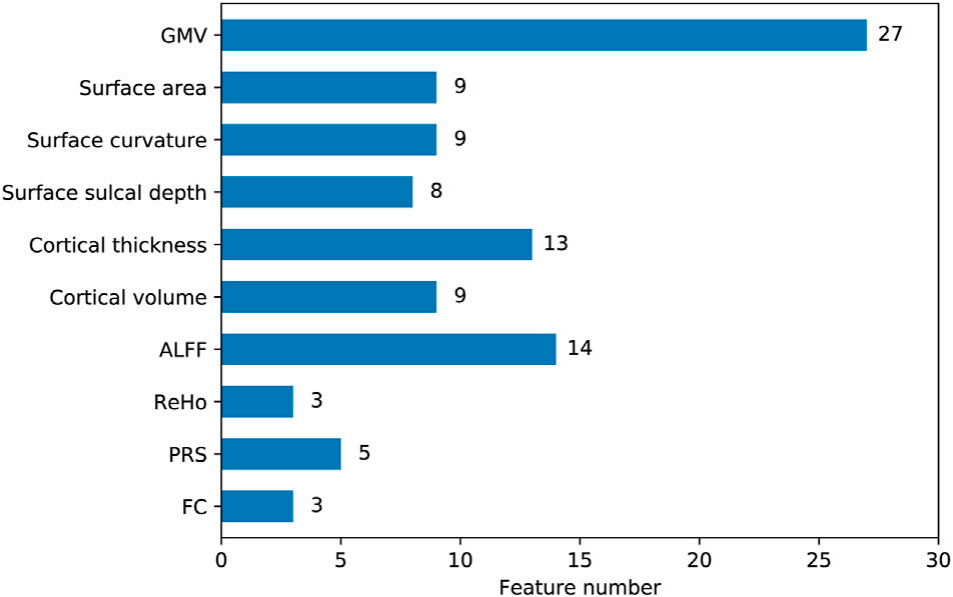
The number of features belonged to each category among the top 100 important features. The y axis represented feature categories. The values labeled on the right of the bars were actual feature numbers.

To quantify feature importance, we selected the classifier that performed the best on the LOOCV procedure (hyperparameter values of this model were given in Table 2). After retraining the classifier on the whole dataset, we directly obtained the importance score of each feature from the ‘feature_importances_” attribute in the fitted model. Typically, a higher importance score implied the corresponding feature was relatively more important in predictions. Among the top 10 important features, nine features were derived from structural imaging, which involved categories of GMV, cortical thickness, cortical volume, surface sulcal depth, and surface curvature. There was only one functional imaging feature (i.e., ALFF), and no genetic features existed (Table 4). Particularly, the GMV in a certain region of the left inferior frontal gyrus (labeled 31 corresponded to the Brainnetome atlas) ranked the first important. When examining the top 100 important features, all the 10 feature categories were involved (Figure 2). More than half of these 100 features belonged to three categories, which were GMV, ALFF, and cortical thickness containing 27, 14, and 13 features respectively.

**TABLE 4:** Top 10 important features obtained from the XGBoost classifier trained on the whole dataset.

Rank	Feature category	Atlas region number	Description	Importance score
1	GMV	31	IFG_L_6_2	0.04138
2	Cortical thickness	157	PoG_L_4_2	0.03584
3	GMV	14	SFG_R_7_7	0.03205
4	ALFF	119	PhG_L_6_6	0.03048
5	Cortical thickness	42	OrG_R_6_1	0.03028
6	Cortical volume	189	MVOcC _L_5_1	0.02930
7	GMV	15	MFG_L_7_1	0.02723
8	Surface sulcal depth	210	LOcC _R_2_2	0.02637
9	Surface curvature	152	PCun_R_4_3	0.02594
10	Surface curvature	169	INS_L_6_4	0.02591

IFG, inferior frontal gyrus; PoG, postcentral gyrus; SFG, superior frontal gyrus; PhG, parahippocampal gyrus; OrG, orbital gyrus; MVOcC, medioventral occipital cortex; MFG, middle frontal gyrus; LOcC, lateral occipital cortex; Pcun, precuneus; INS, insular gyrus. L (R), left (right) hemisphere. The atlas region number corresponded to the Brainnetome parcellation (Fan et al., 2016).

Besides, we further evaluated the prediction contributions of each feature category. In brief, after iteratively removing one feature category, we built XGBoost classifiers with the remaining features following the main analyses to investigate how the performance changed. We found removing any one of these 10 feature categories could lead to a performance drop (Table 5). Specifically, four quantitative metrics including accuracy, sensitivity, F1-score, ROC-AUC decreased consistently, in which the sensitivity measure dropped the most with an average of 21.5%. The specificity had a slight increase (at most 5.4%) in three of the 10 classifiers, indicating a higher bias existed in the three models. In terms of accuracy, the categories of GMV, ALFF, and surface curvature contributed the most to predictions, given removing one of these three categories led to a maximum drop in accuracy score (10.5%). The cortical volume was the least important, since removing this category caused a minimal accuracy decrease (1.8%). PRS exhibited medium importance, excluding of which led to a modest accuracy drop (8.8%).

**TABLE 5:** Prediction performance of classifiers trained with features after removing certain categories.

Feature categories used	Number of features	Accuracy (%)	Sensitivity (%)	Specificity (%)	F1-score (%)	ROC-AUC
No GMV	1737	75.44	65	81.08	65	0.73
No surface area	1773	77.19	65	83.78	66.67	0.74
No surface curvature	1773	75.44	60	83.78	63.16	0.72
No surface sulcal depth	1773	78.95	65	86.49	68.42	0.76
No cortical thickness	1773	80.70	65	89.19	70.27	0.77
No cortical volume	1773	84.21	70	91.89	75.68	0.81
No ALFF	1737	75.44	60	83.78	63.16	0.72
No ReHo	1737	77.19	55	89.19	62.86	0.72
No FC	1933	77.19	60	86.49	64.86	0.73
No PRS	1838	77.19	70	81.08	68.29	0.76

## 4 Discussion

Tremendous evidence has suggested that neuroimaging data coupled with machine learning techniques can provide favorable utilities of prognostic predictions in psychiatric disorders, including schizophrenia (Janssen et al., 2018). A recent relevant study investigated the ranking of predictive capabilities of multiple neuroimaging and clinical measures when predicting the relative change of symptom severity in schizophrenia at 1-year follow-up (Kottaram et al., 2020). The evaluated neuroimaging predictors included structural imaging measures of cortical thickness and gray matter volume as well as functional imaging derived measures of static and dynamic resting-state connectivity. From the aspect of genetic factors, another recent study examined the relationship between polygenic risk scores (PRSs) and antipsychotic drug treatment outcomes in patients with schizophrenia (Zhang et al., 2019). However, it remains unclear 1) whether neuroimaging combined with PRS can provide better prognostic performance than merely using neuroimaging features; and 2) which category of neuroimaging predictors or PRS provides the most accurate prognostic power, and what is the ranking of their importance or contributions. To address these issues, we collected a cohort of patients with schizophrenia (*N* = 57), all of which had baseline neuroimaging and genotype data. All these patients received about 6 weeks of antipsychotic medication treatment. Psychotic symptoms were assessed using PANSS at baseline and follow-up. The patients were grouped into responders or non-responders according to their percentages of PANSS total reduction. We calculated various predictors, including 1) six structural imaging measures (GMV; cortical morphologies of surface area, curvature, sulcal depth, thickness, and volume); 2) three resting-state functional imaging measures (ALFF; ReHo; FC), and 3) step-wise PRS. We trained binary machine learning classifiers with these baseline features to identify whether a patient with schizophrenia was a responder or non-responder.

Overall, we achieved an accuracy of 86% when predicting antipsychotic drug treatment outcomes (i.e., responders or non-responders) of patients with schizophrenia using all feature categories (Table 3). As far as we know, this performance exceeds the vast majority of results in previous studies and is also more than reported in a recent study (Kottaram et al., 2020). The performance was evaluated using a LOOCV procedure, considering our modest sample size (*N* = 57). Although this scheme is supposed to yield unstable estimates of predictive performance (Varoquaux et al., 2017), it is frequently employed in numerous neuroimaging studies, especially in those with relatively small sample sizes (Cao et al., 2020; Kottaram et al., 2020). Specifically, in small datasets, LOOCV can provide sufficient data for training compared with other k-fold cross-validation schemes. In addition to accuracy, we found all other estimated classifier metrics were also at a relatively higher level (Table 3), such as sensitivity (85%), specificity (86%), F1-score (81%), ROC-AUC (0.86) (Figure 1). These extra quantifications further demonstrated our classifier was stable and not biased to predicting either responder (*N* = 20) or non-responder (*N* = 37).

We examined the top 10 important features in predictions and found nine of them were structural imaging measures, including three GMV, two cortical thickness, two surface curvature, one cortical volume, and one surface sulcal depth, one was functional imaging measure of ALFF (Table 4). PRS features were not of top 10 importance. The three GMV features were all extracted from the frontal lobe regions, including inferior, superior, and middle frontal gyri. Particularly, GMV in the inferior frontal gyrus was the most prominent predictor. Previous studies have revealed GMV reductions in the frontal lobe regions were associated with poor antipsychotic medication treatment in patients with schizophrenia (Staal et al., 2001; Quarantelli et al., 2014; Tarcijonas and Sarpal, 2019). Consistently, significant reductions of GMV in the superior and middle frontal gyri were observed in non-responders (Quarantelli et al., 2014). The two cortical thickness features were estimated from the postcentral gyrus in the parietal lobe and the orbital gyrus in the frontal lobe. However, these two regions were discrepant with prior reported regions of the occipital gyrus (Szeszko et al., 2012) and the dorsolateral prefrontal cortex (Zugman et al., 2013). The remaining five features were barely investigated in similar studies, which covered regions of the left parahippocampal gyrus (ALFF), left medioventral occipital cortex (cortical volume), right lateral occipital cortex (surface sulcal depth), right precuneus, and left insular gyrus (surface curvature). When focusing on the top 100 significant predictors, we found all feature categories were involved (Figure 2). Particularly, the top three categories that contained the most features were GMV, ALFF, and cortical thickness, comprising 27, 14, and 13 features respectively. Thus it was straightforward to explain the results that excluding GMV or ALFF features caused the most performance drop of accuracy (10.5%; Table 5). Notably, removing surface curvature features also led to the maximum decrease of accuracy (i.e., 10.5%). Collectively, we considered that GMV, ALFF, and surface curvature features had relatively higher prognostic utilities compared to other feature categories. We observed that removing PRS features gave rise to a modest accuracy drop (8.8%), which was not the least decrease (1.8%) among all categories. This pointed out that PRS features can provide extra prognostic power combined with MRI features, and yet their importance or contributions were between minimum and maximum, inferior to certain MRI measures such as GMV, ALFF, and surface curvature.

There were a few considerations when dealing with predictors and clinical outcomes. We prepared various MRI features, aiming to cover as many measures as possible that were reported in prior relevant studies. We assumed that combining these features would be of great benefit to prognostication since each identified measure could provide certain prognostic information. In our study, although nine MRI measures were computed, more than any previous study used, some were still needed to be examined. For example, the dynamic resting-state functional connectivity measure within the default mode network was demonstrated as the most single accurate predictor of symptom severity change in schizophrenia (Kottaram et al., 2020). As for PRS calculation, it is known that the *p*-value threshold is critical given that only those SNPs with a GWAS association *p*-value below the threshold are included in the procedure (Choi et al., 2020). To avoid potential thresholding effects and duplication of SNPs, 145 step-wise PRSs were calculated as in our previous study (Hu et al., 2021). We defined patients with schizophrenia as responders or non-responders based on their reductions of PANSS total score, which is commonly applied in current practice (Leucht et al., 2009; Cao et al., 2020). However, this approach only focuses on the relative change of PANSS total scores between follow-up and baseline but ignores the actual symptom severity, which can not reflect a clinically significant change. For example, a patient remains highly symptomatic even achieving a 50% reduction of PANSS total score from 120 to 60. Thus it is necessary to further assess whether our features are prognostic of symptom severity (above or below a clinically meaningful cut-off) at follow-up. Another problem is the selection of threshold values, which determines whether a patient is a responder or non-responder. We chose a threshold of 50% in the study, which indicates a much-improved condition for acute patients (Leucht et al., 2009). Different thresholds were proved crucial to clinical trials (Leucht et al., 2007). Therefore, future studies should evaluate prognostications for non-thresholded (i.e., regression analyses) or various fine-step thresholds of PANSS total reductions.

Several limitations need to be considered. First, our sample of patients with schizophrenia was limited for machine learning algorithms, especially for the powerful XGBoost technique (Chen and Guestrin, 2016), which contains more hyperparameters than simple methods such as support vector machines. Although we applied a rational cross-validation strategy, the danger of overfitting can not be eliminated (Varoquaux et al., 2017; Varoquaux, 2018). Larger independent sample replication is required to evaluate the generalizability of our methods. Second, our MRI measures were all calculated based on the Brainnetome atlas (Fan et al., 2016). The choice of brain atlases should not be arbitrary, since it could lead to different results such as in discrimination analysis (Zang et al., 2021). Although we employed a fine-grained parcellation, which contains information on both anatomical and functional connections, comparisons between various brain atlases need to be accomplished. Third, our prediction study just focused on PANSS total reduction, however, it is essential to investigate whether reductions of PANSS subscales (i.e., positive, negative, and general psychopathology) or even specific symptom dimensions could be predicted.

## 5 Conclusion

Polygenic risk score for schizophrenia can provide certain prognostic power when combined with neuroimaging features to predict 6 weeks of antipsychotic medication treatment outcomes in patients with schizophrenia. The relative importance of the polygenic risk score in predictions is between maximum and minimum, lagging behind some neuroimaging measures such as gray matter volume, the amplitude of low-frequency fluctuation, and surface curvature. Overall, our findings inform contributions of the polygenic risk score in machine learning studies that aim to predict treatment outcomes in schizophrenia.

## Data Availability

All the neuroimaging features, polygenic risk scores, and codes used in the study are publicly available at https://github.com/BingLiu-Lab/predict_treatment_outcome_schizophrenia. The raw genotype data are not publicly available but can be obtained by interested researchers upon official request and ethical approval by contacting the corresponding author.
